# Blood pressure-lowering effects of a Bowman-Birk inhibitor and its derived peptides in normotensive and hypertensive rats

**DOI:** 10.1038/s41598-020-66624-3

**Published:** 2020-07-15

**Authors:** Maria Alzira Garcia de Freitas, Nathalia Oda Amaral, Alice da Cunha Morales Álvares, Sandriele Aires de Oliveira, Azadeh Mehdad, Diego Elias Honda, Amanda Sá Martins Bessa, Marcelo Henrique Soller Ramada, Lara Marques Naves, Carolina Nobre Ribeiro Pontes, Carlos Henrique Castro, Gustavo Rodrigues Pedrino, Sonia Maria de Freitas

**Affiliations:** 1 Biology Institute, Department of Cell Biology, Laboratory of Biophysics, University of Brasília (UnB), Quadra 604, Asa Norte, Bloco J 1° andar, Brasília, DF 70910-900 Brazil; 2 Center of Neuroscience and Cardiovascular Physiology; Department of Physiological Sciences, Biological Sciences Institute, Federal University of Goiás, Goiânia, GO 74690-900 Brazil; 3 Integrative Laboratory of Cardiovascular and Neurological Pathophysiology; Department of Physiological Sciences, Biological Sciences Institute, Federal University of Goiás, Goiânia, GO 74690-900 Brazil; 4 Graduate Program in Genomic Science and Biotechnology, and Graduate Program in Gerontology, Catholic University of Brasília, Brasília, DF 70790-160 Brazil

**Keywords:** Biophysical chemistry, Hypertension

## Abstract

Bioactive plant peptides have received considerable interest as potential antihypertensive agents with potentially fewer side effects than antihypertensive drugs. Here, the blood pressure-lowering effects of the Bowman-Birk protease inhibitor, BTCI, and its derived peptides, PepChy and PepTry, were investigated using normotensive (Wistar-WR) and spontaneously hypertensive rats (SHR). BTCI inhibited the proteases trypsin and chymotrypsin, respectively, at 6 µM and 40 µM, a 10-fold greater inhibition than observed with PepTry (60 µM) and PepChy (400 µM). These molecules also inhibited angiotensin converting enzyme (ACE) with IC_50_ values of 54.6 ± 2.9; 24.7 ± 1.1; and 24.4 ± 1.1 µM, respectively, occluding its catalytic site, as indicated by molecular docking simulation, mainly for PepChy and PepTry. Gavage administration of BTCI and the peptides promoted a decrease of systolic and diastolic blood pressure and an increase of renal and aortic vascular conductance. These effects were more expressive in SHR than in WR. Additionally, BTCI, PepChy and PepTry promoted coronary vasodilation and negative inotropic effects in isolated perfused hearts. The nitric oxide synthase inhibitor blunted the BTCI and PepChy, with no cardiac effects on PepTry. The findings of this study indicate a therapeutic potential of BTCI and its related peptides in the treatment of hypertension.

## Introduction

Cardiovascular diseases (CVDs) are one of the leading causes of mortality ^1,2^. Most of the deaths are attributed to stroke, myocardial infarction, coronary artery diseases and atrial fibrillation^3^, in which hypertension and obesity are considered as major risk factors^4^. Although hypertension was identified in the late 1950s as a primary risk factor, it still represents a global public health challenge^1,2^. Thus, it is important to identify novel molecules with therapeutic potential for the treatment of hypertension and other CVDs. Pathophysiological characteristics of hypertension comprise increased total peripheral resistance^5^, endothelium dysfunction^6^ and decreased vascular blood flow^7^.

Plant protease inhibitors are of biotechnological importance and offer pharmaceutical potential for several diseases, including cardiovascular and autoimmune diseases, cancer, and inflammatory processes^8–12^. Protease inhibitors, especially from the Kunitz and Bowman-Birk family (BBI), are commonly found in high quantities in leguminous seeds such as soybean, pea and bean. The black-eyed pea trypsin/chymotrypsin inhibitor (BTCI) - the object of the present study - is a member of the BBI family originally isolated from *Vigna unguiculata* (Cowpea) seeds^13^. BTCI is a small protein of low-molecular mass (9071 Da), with seven disulfide bonds responsible for both the remarkable stability of the protein and theappropriate conformation of two opposite β-hairpins containing reactive sites (K26 and F53) against trypsin and chymotrypsin, respectively^14,15^. This conformation enables the simultaneous and independent inhibition of trypsin and chymotrypsin, driven by an entropic and endothermic process^16–20^. BTCI has been characterized as an enhancer of guanylin-induced natriuresis in isolated rat kidney assays protecting its degradation via chymotrypsin-*like* proteases. BTCI also increases urine flow, Na^+^ excretion and glomerular filtration rate^21^. Intravenous administration of BTCI on Wistar rats has also showna similar effect on bradykinin cleavage, inhibiting plasma serine proteases, as well as enhanced renal aortic vasodilation induced by bradykinin. The BTCI-Bradykinin complex has also been reported to induce smooth muscle contraction in dose-dependent manner^22^.

Antihypertensive agents such as thiazides, β blockers, angiotensin converting enzyme inhibitors, angiotensin receptor antagonists and calcium channel blockers are currently used for blood pressure control in the context of heart attacks and stroke prevention^23^. These antihypertensive drugs, however, can cause adverse side effects, decreasing the effectiveness of drug treatment^24^. In this context, interest has grown in the development of alternative beneficial natural and food products without the side effects observed with antihypertensive drugs^25–27^. Indeed, the nutraceutical potential and pharmacological properties of bioactive peptides from plants and synthetic peptides are widely explored for hypertension treatment^26–31^.

In the present study, the synthetic cyclic peptides, named as PepChy and PepTry, were designed to contain the same amino acid sequence of the two reactive loops present in the BTCI tridimensional structure^16^, each one connected by a disulfide bond. The cyclic peptides contained the same reactive sites of BTCI (K26 and F53), which are responsible for inhibiting trypsin and chymotrypsin, respectively, as well as trypsin-*like* and chymotrypsin-*like* proteases. Recently, a tridimensional structure of PepTry in complex with trypsin was solved at 1.15 Å resolution (PDB code 6EAT)^32^. Similar to BTCI (PDB code 2G81)^19^, this complex is mainly formed by electrostatic interactions involving the lysine reactive site of the PepTry. It is noteworthy that the cyclic peptides were also chosen based on the findings of our previous studies on the characterization of BTCI, as follows: *i)* enhancement of guanylin-induced natriuresis by inhibiting its degradation through chymotrypsin-*like* proteases in isolated rat kidneys^21^; *ii)* anticarcinogenic action in breast cancer cells through inhibition of trypsin-*like*, chymotrypsin-*like* and caspase-*like* proteases of the proteasome 20 S, on invasive MDA-MB-231 breast cancer cells and noninvasive MCF-7 breast cancer cells, as well as presenting no effect on mammary epithelial MCF-10A cells^11,33^; *iii)* potential antihypertensive agent by hemodynamic and cardiovascular effects and its protective action on proteolytic degradation of bradykinin and derived peptides in Wistar rats, via intravenous administration, attributed to its ability to inhibit trypsin-*like* and chymotrypsin-*like* proteases^22^.

Considering the widely documented health benefits of Bowman-Birk inhibitors^9–12,21,22,33–37^, as well as the general potential advantages of bioactive peptides from plants^26–31^ in terms of biodistribution, drug delivery of synthetic peptides, reduced side effects, high specificity and broad spectrum activity, the effects of the Bowman-Birk inhibitor BTCI and its derived peptides on blood pressure were investigated in the present study using normotensive and spontaneously hypertensive rats.

## Results

### Purification of BTCI and derived peptides PepTry and PepChy

**Figure 1:**
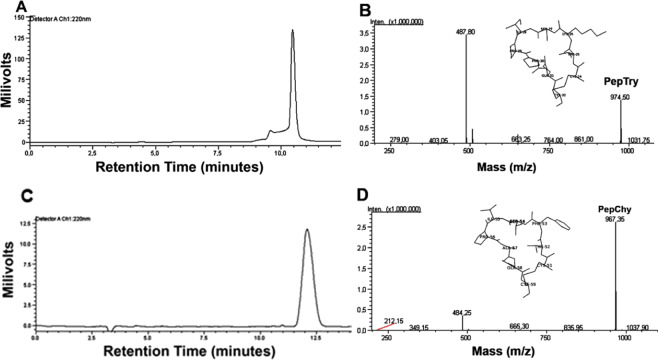
Purification and purity of synthetic peptides PepTry and PepChy. **(A,C)** Reverse-phase chromatography of PepTry and PepChy, respectively, on a C18 Shim-pak VP-ODS column using a linear gradient (5–95%) of acetonitrile. PepTry and PepChy were eluted at 10.5 minutes and 12.0 minutes and 50% and 55% ACN, respectively. **(B**,**D)** EIS-MS spectrometry analysis of PepTry (molecular mass of 974.5 Da) and PepChy (molecular mass of 967.35 Da), respectively. *In sets*: structures of PepTry and PepChy from crystal structure of BTCI^19^ (PDB code 2G81).

Purification of BTCI from *V. unguiculata* seeds, and synthetic peptides derived from the BTCI structure, PepTry (CTKSIPPQC-OH; S-S on Cys 1–9) and PepChy (CTFSIPAQC-OH; S-S on Cys 1–9), and their purity analyses were performed prior to *in vivo* and *ex vivo* assays. As previously reported, BTCI was purified and presented a high purity and molecular mass of 9071.6 Da^13–15,20^. Peptides were chemically synthesized and purified by semi-preparative high performance liquid chromatography (HPLC), as shown in Fig. 1A,C. The RP-HPLC profiles of peptides collected at 10.5 minutes (PepTry) and 12.0 minutes (PepChy) are shown in Fig. 1A,C, respectively. The molecular mass of PepTry (974.5 Da) and PepChy (967.35 Da) and their purities were confirmed by ESI-MS spectrometry, respectively, as indicated by a single spectrum obtained for each peptide (Fig. 1B,D).

### Inhibition assay of BTCI, PepTry and PepChy against trypsin and chymotrypsin

**Figure 2:**
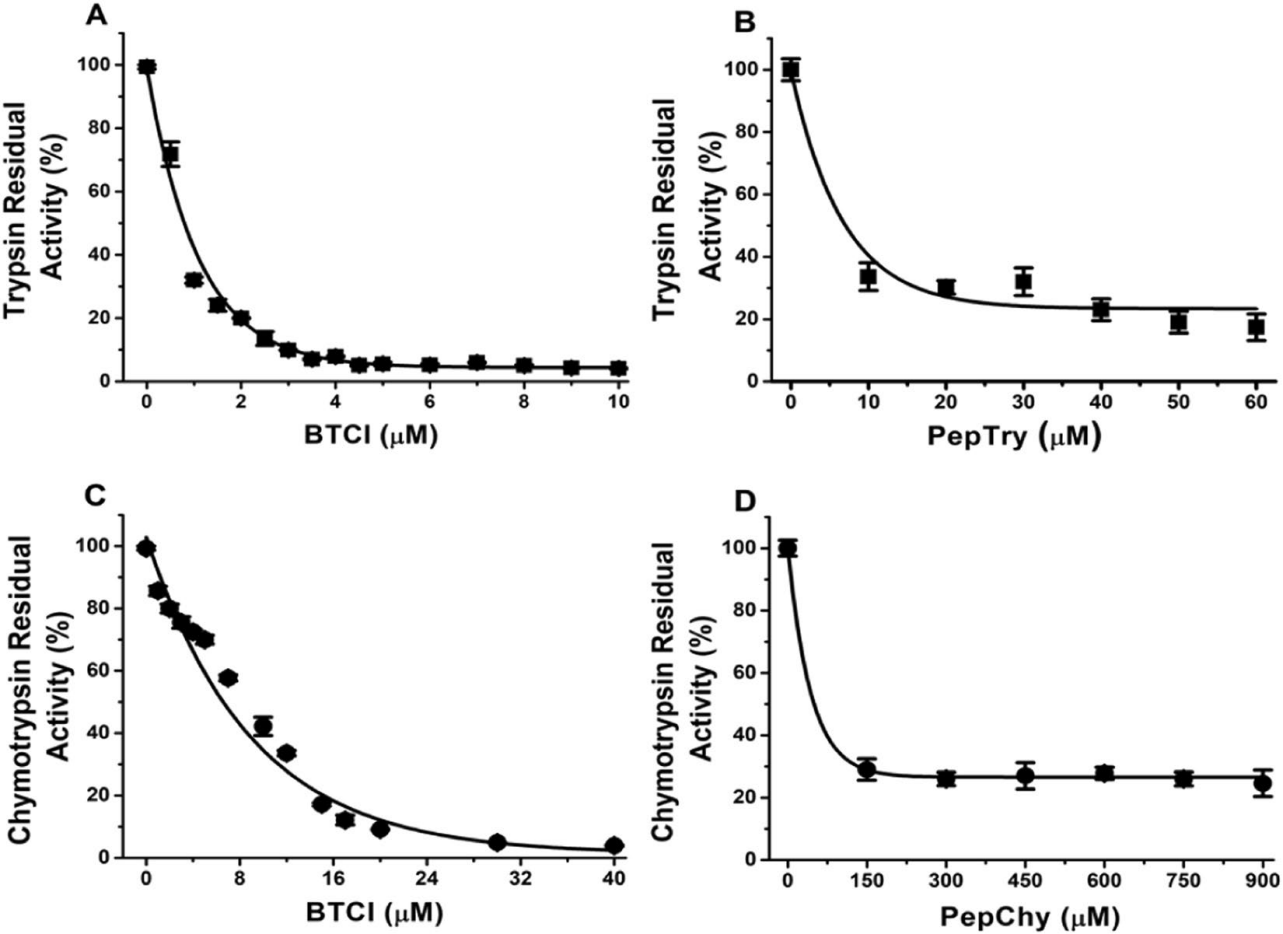
Inhibitory activities of BTCI and its derived peptides. **(A)** Residual activity of trypsin in the presence of increasing concentrations of BTCI and **(B)** PepTry. **(C)** Residual activity of chymotrypsin in the presence of increasing concentrations of BTCI and PepChy **(D)**. The inhibitory assay showed that PepTry and PepChy required a concentration approximately six and eight times higher than BTCI in order to result in total inhibition of trypsin and chymotrypsin, respectively.

The inhibitory activities of BTCI and its derived peptides, PepTry and PepChy, were evaluated through the residual enzymatic activity of serine proteases trypsin and chymotrypsin in the presence of increasing concentrations of BTCI and its derived peptides (Fig. 2A–D). In the absence of BTCI and peptides, the proteases cleave BAPNA/GPNA substrates releasing the yellow-colored p-nitroanilide product corresponding to an enzymatic activity of 100%^39^. In the presence of BTCI and peptides, a gradual decay of the protease activities is observed. BTCI at approximately 6 µM and 40 µM inhibits trypsin and chymotrypsin, respectively, a 10-fold greater inhibition than for PepTry (60 µM) and PepChy (400 µM). The inhibition constants, Ki, of BTCI and PepTry against trypsin were 0.59 ± 0.03 × 10^−8^ M^16^ and 1.19 ± 0.04 × 10^−7^ M, respectively; Ki values against chymotrypsin for BTCI and PepChy were 1.15 ± 0.04 × 10^−7^ M^17^, and 1.21 ± 0.26 × 10^−4^ M, respectively. These values indicate that BTCI is a more potent inhibitor against trypsin than chymotrypsin, with PepTry more potent than PepChy.

**Figure 3:**
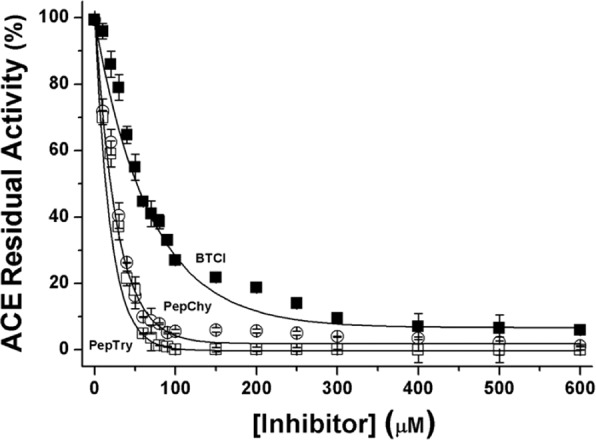
Inhibitory activities of BTCI (■—■), PepChy (ο—ο) and PepTry (□—□) against angiotensin converting enzyme (ACE). All molecules present inhibition closest to 95%. IC_50_ values were estimated for BTCI, PepChy and PepTry at 54.6 ± 2.9 µM, 24.7 ± 1.1 µM, and 24.4 ± 1.1 µM, respectively.

In the present study, the ACE-inhibitory activity of BTCI and its derived peptides, PepChy and PepTry was evaluated at different concentrations (0–600 µM) as shown in Fig. 3. The ACE-inhibitory activity increased in a dose-dependent pattern. The highest inhibitory value of about 98% was recorded for PepTry and PepChy at 100 µM, whereas approximately 300 µM of BTCI was required to reach 94% of ACE-inhibition. The ACE-inhibition curve fitting was conducted by a non-linear method, from which the IC_50_ values for BTCI, PepChy and PepTry were estimated as 54.6 ± 2.9 µM, 24.7 ± 1.1 µM, and 24.4 ± 1.1 µM, respectively.

**Figure 4:**
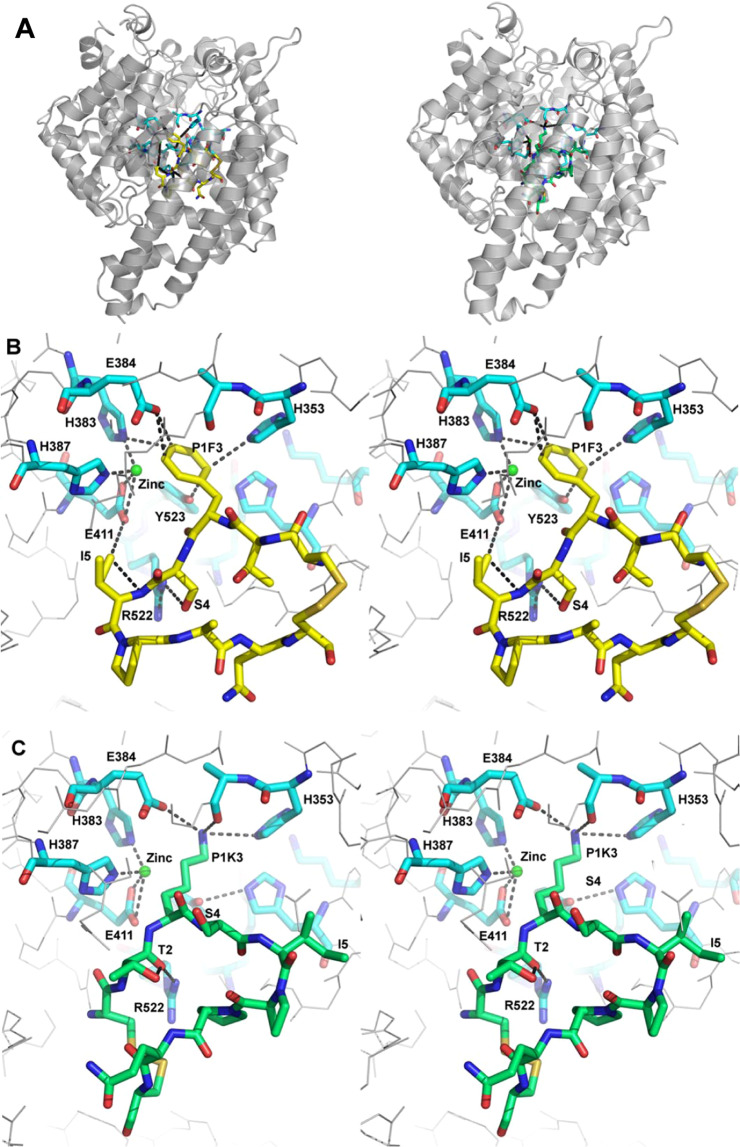
Tridimensional structures of the ACE-PepChy and ACE-PepTry complexes obtained by docking. **(A)** ACE (gray cartoon) in complex with PepChy (yellow sticks) and PepTry (green sticks). **(B)** Stereo view (cross-eyed) of ACE active site (blue) with bound PepChy (yellow). Interacting residues of the ACE active site and reactive site of PepChy (P1F3) are labelled. Atoms are coloured as red for oxygen, blue for nitrogen and green sphere for zinc ion. Hydrogen bonds at the complex interface are shown as dotted lines. **(C)** Stereo view (cross-eyed) of ACE active site (blue) with bound PepTry (green). Interacting residues of the ACE active site and reactive site of PepTry (P1K3) are labelled. Atoms are coloured as red for oxygen, blue for nitrogen and green sphere for zinc ion. Hydrogen bonds at the complex interface are shown as dotted lines. The crystallographic structures used by docking procedure were: ACE PDB code 1O8A^40^, PepTry and PepChy from BTCI^19^ (PDB code 2G81).

The ACE-inhibitory effects of PepChy and PepTry are consistent with molecular docking simulation. This methodology was used to predict the interactions of the inhibitors PepChy and PepTry with ACE by calculating interaction energies for the best pose of inhibitors closest to catalytic site of the enzyme. Best poses were achieved for both peptides, which occlude the enzyme catalytic pocket (Fig. 4A–C), interacting with **ΔG** binding energies of about -11.0 kcal/mol. The majority of hydrogen bonds were observed between PepChy and ACE (Fig. 4B), involving three amino acid residues in PepChy (F3, S4 and I5) and six amino acid residues in ACE (R522, E411, Y523, H383, E384 and H353). For PepTry, the majority of hydrogen bonds were observed between two amino acid residues in PepTry (T2 and K3) and three amino acid residues in ACE (R522, E384 and A354). Moreover, electrostatic interaction was observed between PepTry (K3) and ACE (E384), with T-shapedπ-π-interactions between phenyl rings of Y523 in ACE and F3 in PepChy. It is noteworthy that amino acid residues H387, H383, E384 and E411 involved in coordination of Zinc in the native ACE active site also interact with PepChy and PepTry (Fig. 4B,C). Overall, the results showed that the interactions between ACE and the peptides occur within the catalytic site, leading to the complete inhibition of the enzyme, corroborating with the estimated IC_50_, as described above (Fig. 3).

### *In vivo* assays

**Figure 5:**
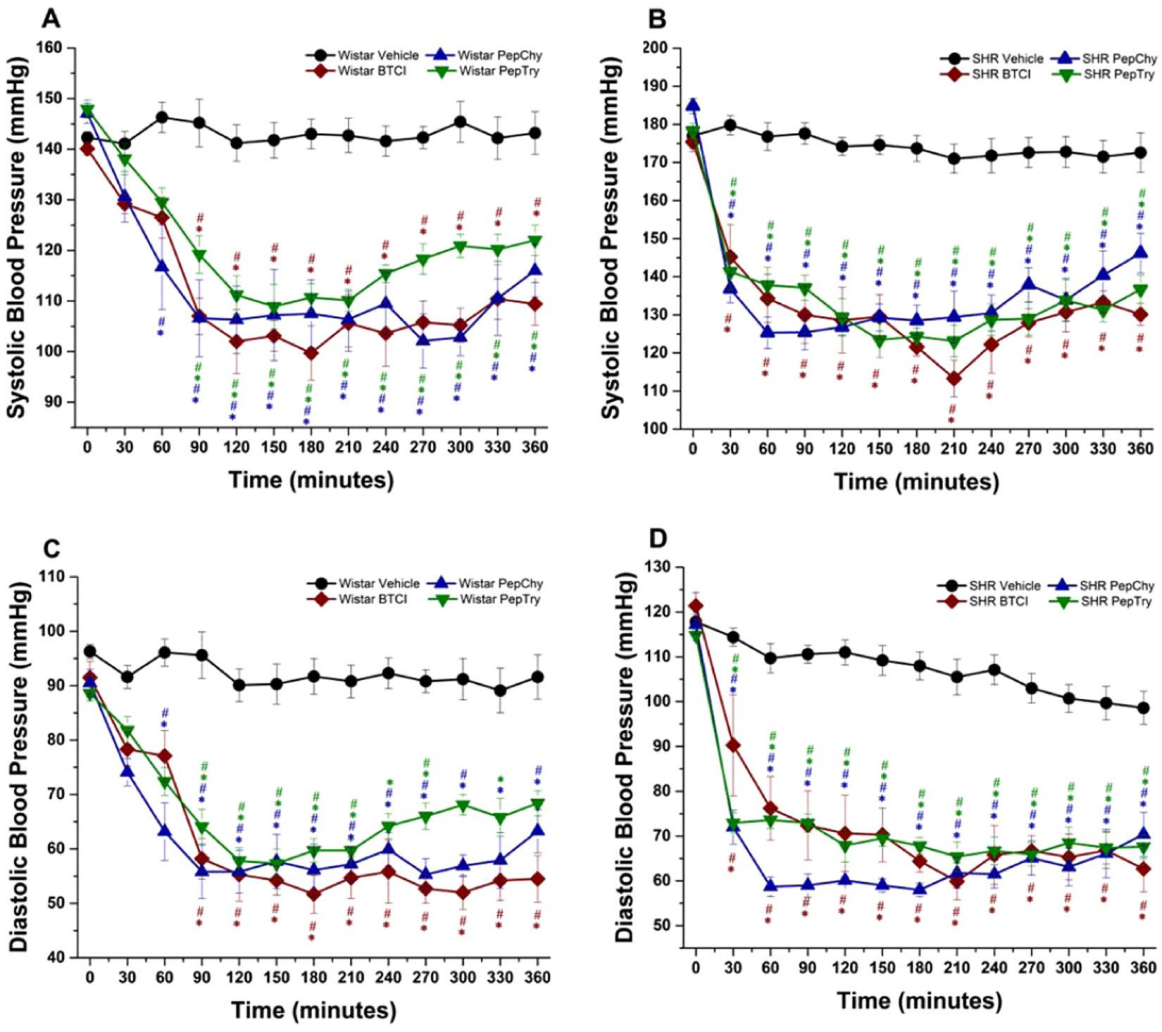
Hemodynamic effects in WR and SHR induced by gavage administration of BTCI (30.0 mg·kg^−1^), PepTry (3.3 mg·kg^−1^) and PepChy (3.3 mg·kg^−1^) or Vehicle (NaCl 0.9%), in anesthetized rats. Systolic blood pressure in **(A)** Wistar and **(B)** SHR; Diastolic blood pressure in **(C)** Wistar and **(D)** SHR. The results are expressed as the mean ± SEM. *p < 0.05 compared to vehicle; ^# ^p < 0.05 compared to basal time.

**Figure 6:**
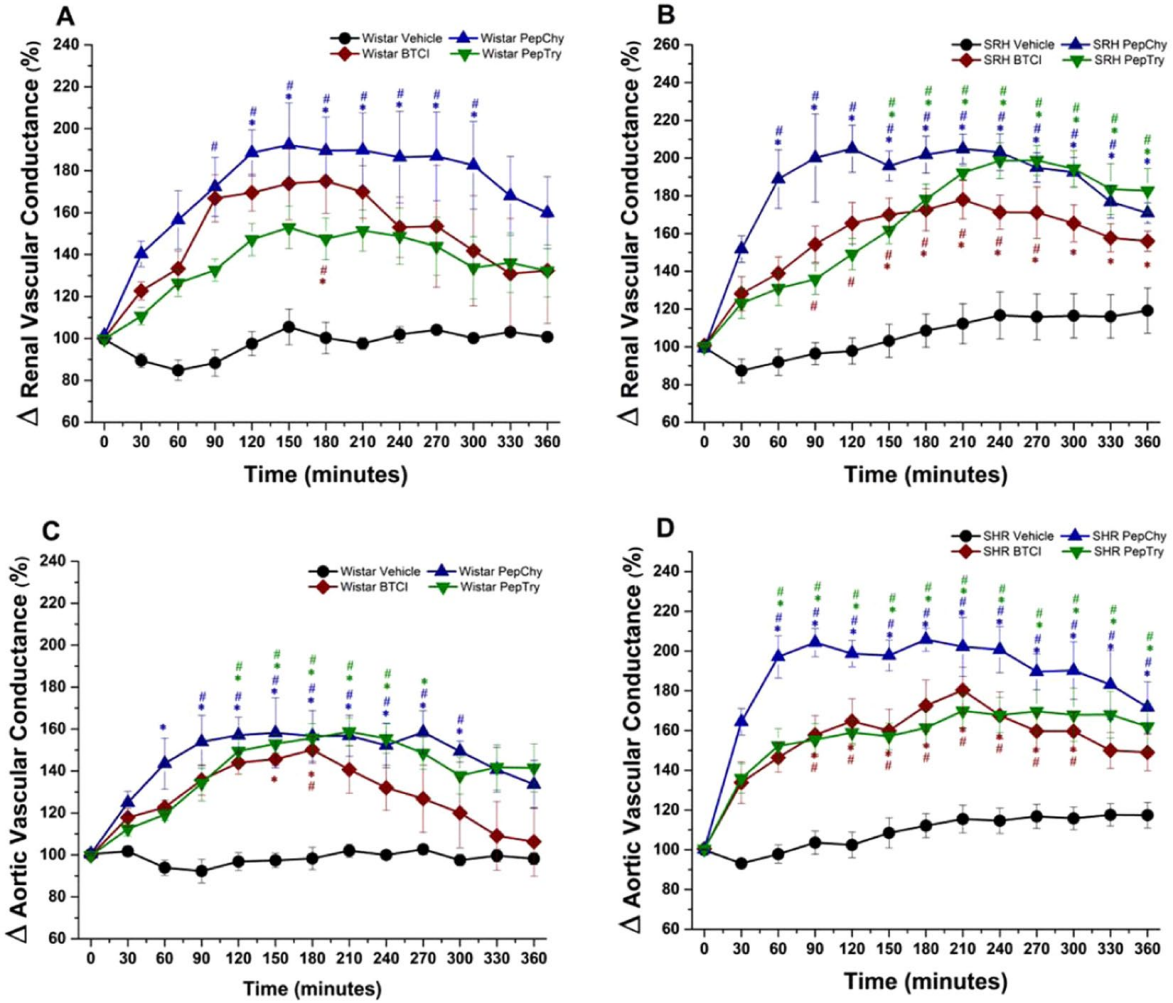
Cardiovascular responses induced by gavage administration of BTCI (30.0 mg·kg^−1^), PepTry (3.3 mg·kg^−1^) and PepChy (3.3 mg·kg^−1^) or Vehicle (NaCl 0.9%), in anesthetized rats. Renal vascular conductance in **(A)** Wistar and **(B)** SHR; Aortic vascular conductance in **(C)** Wistar and **(D)** SHR. The results are expressed as the mean ± SEM. *p < 0.05 compared to vehicle; ^#^p < 0.05 compared to basal time.

**Figure 7:**
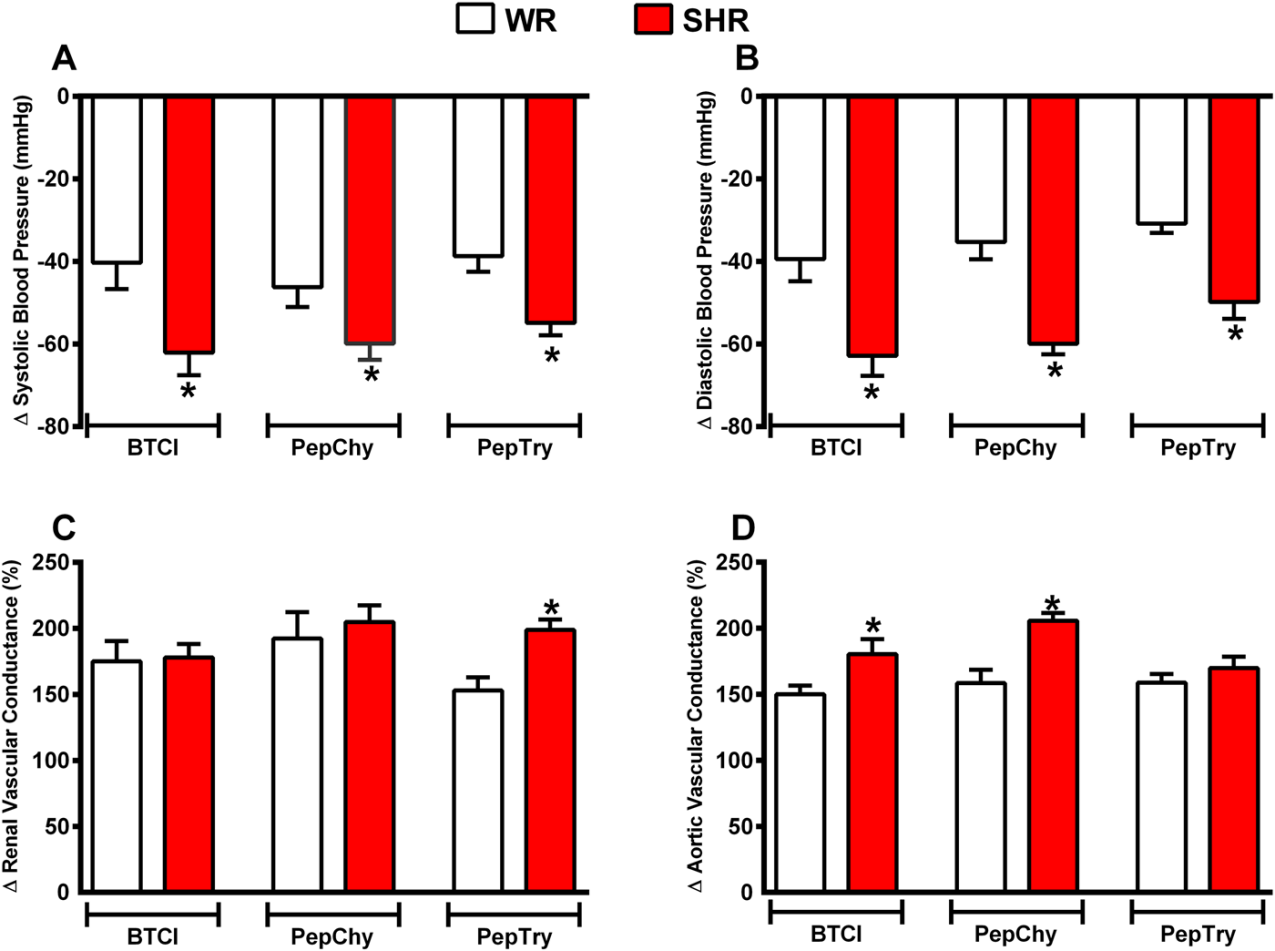
Maximum response induced by gavage administration of BTCI (30.0 mg·kg^−1^), PepTry (3.3 mg·kg^−1^) and PepChy (3.3 mg·kg^−1^) in anesthetized WR rats (n = 6) and SHR (n = 6). **(A)** Systolic blood pressure; **(B)** Diastolic blood pressure; **(C)** Renal vascular conductance; **(D)** Aortic vascular conductance. The results are expressed as the mean ± SEM. *p < 0.05 compared to WR..

Cardiovascular and hemodynamic effects were evaluated in normotensive (Wistar, WR) and spontaneously hypertensive rats (SHR). Equimolar concentrations (3.31 mM) were employed for all molecules, with doses at 30.0 mg/kg for BTCI, 3.3 mg/kg for PepTry and PepChy), and 0.9% for NaCl (Vehicle) (Figs. 5A–D, 6A–D, 7A–D). As expected, the vehicle did not promote significant changes in any of the hemodynamic parameters. In contrast, all cardiovascular parameters were modified after treatment with BTCI and its derived peptides (Figs. 5A–D, 6A–D). The comparison of the maximum response to BTCI, PepChy and PepTry between normotensive and SHR is presented in Fig. 7A–D.

### Cardiovascular effects of BTCI, PepTry and PepChy administrated via gavage on SBP, DBP, RVC and AVC hemodynamic parameters in WR and SHR rats

BTCI in WR (n = 6) decreased systolic blood pressure (SBP) (140.1 ± 1.3 to 102.0 ± 6.3 mmHg; Δ −38.1 mmHg, from the baseline, Fig. 5A) and dyastolic blood pressure (DBP) (91.5 ± 3.0 to 51.7 ± 3.5 mmHg; Δ − 39.8 mmHg, from the baseline, Fig. 5C). Additionally, the drug increased renal vascular conductance (RVC) (72% ± 15.3%, Fig. 6A) and aortic vascular conductance (AVC) (50.1 ± 6.5%, Fig. 6C). In SHR (n = 6), marked decreases were noticeable from baselines for SBP (175.4 ± 2.5 to 113.3 ± 4.8 mmHg; Δ −62.3 mmHg, Fig. 5B) and DBP (121.3 ± 2.7 to 59.9 ± 4.1 mmHg; Δ −61.4 mmHg, Fig. 5D). In addition, increases of RVC (78 ± 10.1%, Fig. 6B) and AVC (80.3 ± 11.5%, Fig. 6D) were achieved. Additionally, Fig. 7 shows the comparison of maximum cardiovascular responses induced by BTCI, PepChy and PepTry between SHR and WR. It is noteworthy that SBP, DBP and AVC variations promoted by BTCI were more expressive in SHR than in WR (Fig. 7A,B,D).

PepTry gavage at a dose of 3.3 mg·kg^−1^ in WR rats (n = 6) resulted in decreases from the baseline for SBP (147.9 ± 1.8 to 108.9 ± 4.4 mmHg; Δ − 39.0 mmHg, Fig. 5A) and DBP (88.6 ± 1.4 to57.3 ± 2.7 mmHg; Δ −31.3 mmHg, Fig. 5C). Increases were also observed for RVC (52.9 ± 10.1%, Fig. 6A) and AVC (58.7% ± 6.6%, Fig. 6C). In SHR (n = 6) decreases from the baseline were observed in SBP (178.3 ± 2.0 to 123.4 ± 4.6 mmHg; Δ −54.9 mmHg, Fig. 5B) and DBP (114.8 ± 1.2 mmHg to 65.4 ± 3.3 mmHg; Δ −49.4 mmHg, Fig. 5D). Increases were observed in RVC (98.8 ± 7.9%, Fig. 6B) and AVC (69.9 ± 8.5%, Fig. 6D). The variations observed in SBP, DBP and RVC promoted by PepTry were more expressive in SHR than in WR (Fig. 7A–D).

PepChy oral gavage at dose of 3.3 mg·kg^−1^ in WR rats (n = 6) decreased both SBP and DBP from the baseline (147.1 ± 2.0 to 106.3 ± 6.7 mmHg; Δ − 40.8 mmHg, Fig. 5A) and (90.5 ± 2.5 to 55.8 ± 3.9 mmHg; Δ−34.7 mmHg, Fig. 5C), respectively. In addition, RVC (92.30 ± 19.9%, Fig. 6A) and AVC (58.2% ± 16.8% Fig. 6C) increased. In SHR (n = 6), the PepChy oral gavage resulted in decreased SBP and DBP from baselines (184.8 ± 2.0 to 126.8 ± 2.3 mmHg; Δ−58.0 mmHg, Fig. 5B) and (117.2 ± 2.2 to 59.0 ± 2.6; Δ−58.2 mmHg, Fig. 5D), respectively. Increases were observed for RVC (105.0 ± 12.5%, Fig. 6B) and AVC (105.8 ± 5.8%, Fig. 6D). As observed for BTCI and PepTry, all hemodynamic responses and cardiovascular parameters in both WR and SHR were significantly altered. In addition, the changes in SBP, DBP and AVC promoted by PepChy were more expressive in SHR than in WR (Fig. 7A–D).

As shown in Figs. 5–7, values of hemodynamic parameters evaluated in WR rats in the presence of BTCI and derived peptides presented reductions of SBP and DBP. In contrast, values for RAC and AVC increased following administration of BTCI and peptides, with the greatest response for BTCI and PepChy at 180 minutes, and PepTry at 270 minutes. In the SHR rats, values of SBP and DBP were similar, with faster reduction for BTCI and PepChy, when compared toPepTry. In contrast, an increase in RAC and AVC hemodynamic parameters was observed after 30 minutes administration of BTCI and peptides, with greater expressiveness for PepChy.

The results presented in Fig. 6A–D indicate significant increases in the renal and aortic conductance for all molecules, up to 180 minutes, compatible with a vasodilation effect on the respective arteries. Indeed, comparing the WR and SHR models studied for BTCI, the measured values were similar up to 180 minutes differing from 180 to 360 minutes. At 360 minutes, the values for the WR rats drastically decreased near to the basal level. These data indicate that vasodilation of the renal artery was greater than for the aortic artery (Fig. 6A,C). The expressive increase of CVR and AVC for PepChy in both WR and SHR models is noteworthy (Fig. 6A–D). Altogether, the *in vivo* assays suggest that BTCI and related peptides resulted in a greater hypotensive effect in SHR than in WR rats (Fig. 7A–D).

### Effects of BTCI, PepChy and PepTry on left ventricular contractility and coronary vasodilation

**Figure 8:**
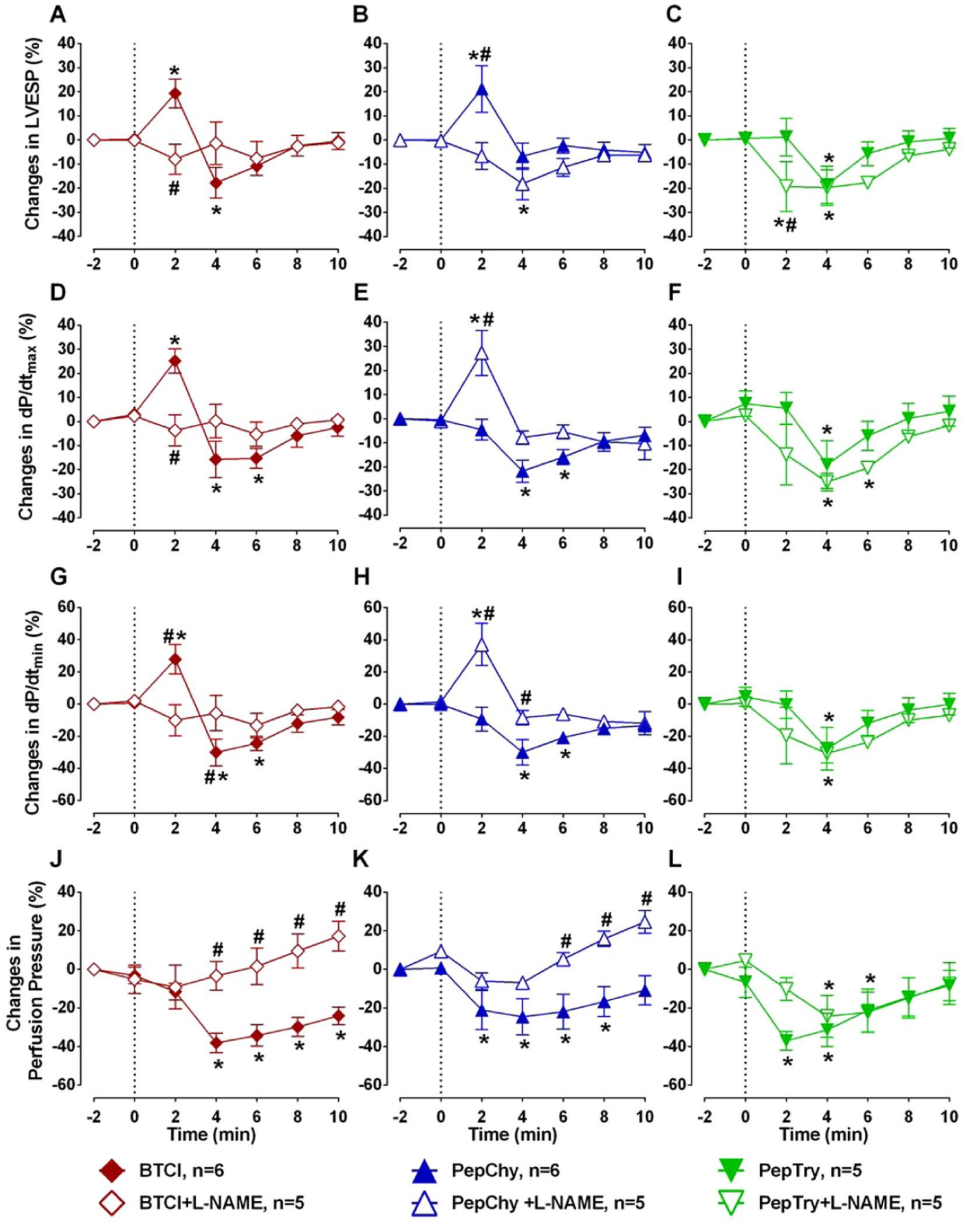
Effects of BTCI, PepChy and PepTry in isolated rat hearts. Effects of the PepChy, BTCI and PepTry in the **(A–C)** Left ventricular end-systolic pressure (LVESP), **(D–F)** Maximal Rate of Left Ventricular Pressure Rise (dP/dt_max_), **(G–I)** Maximal Rate of Left Ventricular Pressure Decline (dP/dt_min_), and **(J–L)** Perfusion Pressure. The dotted line represents the beginning of peptide infusion. Data are reported as mean ± SE. *p < 0.05 vs. baseline, ^#^*p* < 0.05 vs. between time points. *Two-way* ANOVA was followed by Sidak’s multiple comparison post-test.

The effects of BTCI, PepChy and PepTry on left ventricular contractility and coronary vasomotricity in isolated hearts are presented in Fig. 8A–L. All molecules showed expressive changes in left ventricular contractility and coronary vasodilation.

The BTCI infusion promoted an increase followed by a significative reduction in the left ventricular end-systolic pressure (LVESP, E_max_: −17.83 ± 4.34%, p < 0.05) (Fig. 8A). In addition, BTCI promoted a reduction in a maximal rate of left ventricular pressure rise (dP/dt_max_, E_max_: −15.73 ± 5.22%, p < 0.05) and in a maximal rate of left ventricular pressure decline (dP/dt_min_, E_max_: −30.15 ± 7.21%, p < 0.05) (Fig. 8D,G). BTCI also promoted a coronary vasodilation, observed by the reduction of the perfusion pressure (E_max_: −38.08 ± 5.77%, p < 0.05) (Fig. 8J) without altered heart rate (data not shown). Pre-treatment with L-NAME blunted the cardiac effects of BTCI.

PepChy, as observed with BTCI, also induced a significative reduction in the LVESP (E_max_: −18.13 ± 2.38%, p < 0.05), dP/dt_max_ (E_max_: −21.73 ± 3.03%, p < 0.05) and dP/dt_min_ (E_max_: −29.95 ± 4.08%, p < 0.05) (Fig. 8B,E,H). This peptide also promoted a reduction in perfusion pressure (E_max_: −24.59 ± 3.94%, p < 0.05) (Fig. 8K). The pre-treatment whith L-NAME abolished these effects. Unexpectedly, PepChy promoted a transitory increase in LVESP, dP/dt_max_ and dP/dt_min_ and dt/dt_max_ and dP/dt_mi_n in the presence of L-NAME (Fig. 8B,E,H). The heart rate remained unaltered by PepChy (data not shown).

Perfusion with PepTry in the isolated hearts also evoked a significative reduction in the LVESP (E_max_: −18.55 ± 2.70%, p < 0.05), dP/dt_max_ (E_max_: −17.89 ± 3.29%, p < 0.05) and dP/dt_min_ (E_max_: −27.78 ± 4.15%, p < 0.05) (Fig. 8C,F,I). This peptide also induced a significant decrease in the perfusion pressure (E_max_: −37.09 ± 5.12%, p < 0.05) (Fig. 8L). No changes were observed in the heart rate (data not shown). Treatment with L-NAME only attenuated the reduction of the LVESP promoted by PepTry (Fig. 8C), but did not inhibit the effects on dP/dt_max_,dP/dt_min_, or perfusion pressure (Fig. 8F,I,L).

## Discussion

Hypertension is a worldwide health problem, considered a major risk factor for stroke cardiovascular morbidity and mortality, as well as renal diseases. Most classes of antihypertensive drugs, in addition to providing cardiovascular benefits, can prevent non-cardiovascular diseases such as glaucoma and diabetes^23^. Nevertheless, adverse side effects in some patients have been responsible for limitations and reduced compliance of current treatments^23,24,41^. The pharmacological potential of bioactive peptides from plants and synthetic peptides has been widely examined, with activity in number in the treatment of hypertension also explored^26–31^.Continued investigation of novel molecules, as natural, synthetic or modified compounds, has been recommended for drug development with therapeutic potential for treatment of hypertension and other cardiovascular diseases^25,30,31,42^. In the present study, hypotensive and vasodilator properties were investigated in BTCI and two newly synthesized peptides derived from BTCI, namely PepChy and PepTry. Additionally, potential mechanisms of action underlying hypotensive effects were evaluated.

BTCI is well-characterized as a “double-headed” protease inhibitor capable of inhibiting trypsin and chymotrypsin independently and simultaneously, irreversibly blocking the enzyme active site through its two specific reactive sites (P1), K26 and F53, respectively^14,16–19,43,44^. Here, bioactive peptides derived from BTCI were designed to contain these reactive sites for trypsin (PepTry) and chymotrypsin (PepChy). These inhibited the proteases with less affinity than BTCI, as indicated by inhibition constants. Comparatively, the lower affinity of these peptides may be due to the lack of structural regions such as those stabilizing the whole interface of the BTCI-protease complex. This is in agreement with previous reported data in which cyclic peptides from the BBI family with additional amino acid residues after the disulfide bond presented a higher inhibitory activity against proteases^45,46^.

Bioactive peptides have been described as blood-pressure lowering agents by decreasing renin or angiotensin-converting enzyme (ACE) activities, and by regulating the NO synthesis pathway^27,28^. Among these, peptides with high antihypertensive potency have been designed based on the side chain amino acid cleavage position. Some of these containing charged (arginine and/or lysine), hydrophobic (valine, isoleucine, proline) and mainly aromatic amino acids (phenylalanine, tyrosine) residues, were identified as ACE inhibitors and characterized as potent antihypertensive agents^47–49^. A BIOPEP database of biologically active peptides, including the ACE-inhibitors, together with their amino acid sequences, is available at http://www.uwm.edu.pl/biochemia/index.php/pl/biopep ^48^. Nevertheless, to date, no cyclic peptides derived from BBIs such as BTCI, which contain reactive sites formed by aromatic and positive charged amino acid residues, have been investigated as antihypertensive agents.

Similarly to BTCI, the two cyclic peptides studied contain the same reactive sites K26 and F53 responsible for inhibiting trypsin and chymotrypsin, respectively and also trypsin- and chymotrypsin-*like* proteases. These peptides were chosen based on our previous studies, where the natriuretic, hemodynamic, cardiovascular and anticarcinogenic effects of BTCI were attributed to its ability to inhibit trypsin and chymotrypsin-*like* proteases^11,21,22,33^. Therefore, as expected, all results showed that blood pressure-lowering on WR and SHR is associated with the capability of BTCI and both peptides to inhibit proteases, such as ACE, and other trypsin/chymotrypsin-*like* proteases. Therefore, here, the ACE-inhibitory activity of BTCI, PepChy and PepTry increased in a dose-dependent manner, presenting IC_50_ values estimated at 54.6 ± 2.9 µM, 24.7 ± 1.1 µM, and 24.4 ± 1.1 µM, respectively.

The inhibition activities of BTCI and the peptides against ACE was performed in order to investigate the possible mechanism involved in blood pressure-lowering on WR and SHR, given that ACE is a protease involved in blood pressure control and BTCI and tested peptides are proteases inhibitors. As observed, maximum concentration of BTCI for inhibition of ACE were greater (300 µM) compared with trypsin and chymotrypsin inhibition (6 µM and 40 µM, respectively), indicating that BTCI is a more potent inhibitor against trypsin and chymotrypsin than ACE. Therefore, the differences in maximum concentration for inhibition of ACE by BTCI compared to those proteases can be attributed to conformational differences in the active site of ACE, leading to steric impediments that avoid or hinder optimal interaction between BTCI and ACE molecules.

Based on these results, the inhibitory activities of BTCI and peptides against ACE may be mainly due to the presence of the basic (lysine) and the hydrophobic (phenylalanine) amino acids in the reactive loops that also determine the potency of protein and peptide inhibition of trypsin and chymotrypsin proteases. In all three molecules, the positively charged ε-amine group derived from lysine and the phenylalanine side chain might have occluded the ACE active site. It is noteworthy that peptides containing aromatic, positively charged and some hydrophobic residues (proline, isoleucine, valine and leucine), such as BTCI, have been reported as strong ACE-inhibitors and antihypertensive agents^49^. Additionally, the IC_50_ values of BTCI, PepChy and PepTry are in agreement with ACE-inhibitory peptides from several proteins deposited in the BIOPEP database (http://www.uwm.edu.pl/biochemia/index.php/en/biopep)^48^.

The ACE-inhibitory effects of PepChy and PepTry were consistent with molecular docking simulation. As seen in Fig. 4, the hydrophobic phenylalanine and the charged lysine amino acids in all the inhibitors make the most important hydrophobic and electrostatic interactions in the ACE active site and are responsible for inhibiting ACE activity.

The docking simulation showed that the interaction between the two peptides and ACE occurs with the key residues within the catalytic site. This consists of E411, Y523, V518, H353, H513, E384, A354, Y520, E384 and Zn^2+^. The stability of ACE peptides interaction is represented by the binding energy values, dependent on the number of hydrogen bonds, hydrophobic and electrostatic interactions, as well as interaction with Zn^2+^ cofactor. Typically, high stability is related to a better inhibition showing lower IC_50_ value. The best results were obtained for PepChy and PepTry at the ACE active site near to the Zn^2+^ ion. The lowest values of binding energies and IC_50_ of approximately -11.0 kcal/mol and 24.0 µM, respectively, were compatible with the high stability of the ACE-peptide complexes. The peptides bind to the active site pocket of ACE through hydrogen bonds, and hydrophobic and electrostatic forces. In addition, PepChy and PepTry are also linked through F3 and K3, respectively, with specific amino acid side chains responsible for zinc coordination, such as E411, H383 and E384, by induced ion-dipole and electrostatic interactions. It has been reported that the same interactions play an important role in stabilizing the interface of the ACE-lisinopril and selenium analogues of captopril complexes^38,40^. Therefore, these results suggested that these interactions contribute to the stabilization of the ACE-peptide complex, which lead to strong ACE-inhibitory activity. Together, these results highlight the important *in vitro* and *in vivo* antihypertensive properties and potential roles for these molecules in the reduction of blood pressure, as discussed below.

The present study was firstly motivated by the inhibitory effects of BTCI related to its natriuretic effects on the renal system model of Wistar rats. As reported, BTCI enhanced guanylin-induced natriuresis^21^ and showed a protective effect on bradykinin towards plasma serine proteases cleavage as well as enhanced renal aortic vasodilation induced by bradykinin^22^. Furthermore, it is also known that the cyclic peptides with nine amino acid residues stabilised by one present disulfide bond, in addition to their inhibitory activity against trypsin and chymotrypsin, through lysine and phenylalanine reactive sites, respectively, also offer structural rigidity and stability and can be tested and used as a natural protease inhibitor^45,46^. Given the structural and inhibitory properties of BTCI and peptides, as well as the natriuretic and hemodynamic effects of BTCI, the hypotensive action through the peripheral vascular resistance and endothelial nitric oxide synthase (eNOS)/nitric oxide (NO) (eNOS/NO) pathway *in vivo* and *ex vivo*, respectively, were investigated in the present study.

The results of *in vivo* assays showed: I. BTCI and related peptides promoted a decrease of SBP and DBP and renal and aortic vasodilation in WR and SHR; II. The decrease in SBP, DBP and aortic vasodilation promoted by BTCI and PepChy is more expressive in SHR than WR; III. The reduction in SBP, DBP and renal vasodilation caused by PepTry administration is more expressive in SHR than WR. Taken together, the results demonstrate that BTCI and related peptides have antihypertensive and vasodilator effects. These findings highlight the therapeutic potential of the compounds in the treatment of hypertension.

In general, the blood pressure-lowering effects of drugs, chemical compounds or bioactive peptides are related with ability to modulate the renin-angiotensin system by the inhibition of renin or angiotensin-converting enzyme (ACE), as well as due to increased enzymatic activity of the endothelial nitric oxide synthase (eNOS), leading to increased nitric oxide (NO)^27,28^. Antihypertensive drugs to reduce systolic or diastolic blood pressure are important in prevention of cardiovascular diseases and stroke. Our data demonstrate that BTCI and peptides administration promoted a decrease of SBP and DBP and an increase in RVC and AVC in WR and SHR. These increases indicate vasodilation in the renal and aortic territories, which possibly leads to the hypotension observed after oral administration of BTCI and the peptides. All the results are consistent with our previous data, which showed that BTCI has a protective effect on bradykinin cleavage through inhibition of plasma serine proteases, with vasodilation and hypotensive responses after intravenous administration^22^. It has also been demonstrated that BTCI enhanced the effect of guanylin on natriuresis, increasing urine flow, Na^+^ excretion and glomerular filtration rate^21^. In addition, the greatest effects of guanylin on osmolar clearance were achieved in the presence of BTCI. It is noteworthy that the results obtained here are consistent with those reported for bioactive peptides, hydrolysed proteins and commercial compounds that reduce SBP and DBP and increase renal and aortic vasodilation^28,49–51^. Altogether, these effects and the sodium and water/urine excretion (pressure-natriuresis) promoted by BTCI can provide a compensatory effect on reducing blood pressure by decreasing the peripheral vascular resistance^52–54^.

PepTry and PepChy also showed vascular relaxation and consequent hypotensive effects *in vivo*. We highlight the PepChy effects, since this peptide promoted expressive aortic vasodilation in SHR. Additionally, we demonstrated that hypotensive responses observed by peptide administration were more expressive in SHR than in WR. Therefore, these results indicate a possible therapeutic potential of BTCI and its related peptides in the treatment of hypertension.

The renin-angiotensin system (RAS) plays a crucial role in regulating blood pressure and electrolyte homeostasis. ACE catalyzes the degradation of the blood pressure lowering bradykinin that is responsible for the release of prostacyclin and nitric oxide (NO) on blood vessels^40,55–58^. These systems were tested in *ex vivo assays* only for BTCI and PepChy, considering their higher expressive hypotensive effects *in vivo* compared to PepTry. Interestingly, no inhibitory effects of BTCI and its derived peptide, PepChy, were detected in the specific systems.

Nevertheless, BTCI and these peptides were able to promote an important coronary vasodilation, observed by the reduction in the perfusion pressure of the isolated hearts. These finding are also in agreement with the increase in the vascular conductance observed in *in vivo* assays. In addition, coronary vasodilation induced by BTCI or PepChy was completely blunted by the nitric oxide synthase inhibition. Pre-treatment with L-NAME, however, did not change the reduction in perfusion pressure induced by PepTry. Therefore, the coronary vasodilation induced by BTCI and PepChy is mediated by the eNOS/NO pathway regulating the vascular tonus of the coronary arteries and myocardial contractility^59–64^. In contrast, the coronary vasodilation induced by PepTry appears to be mediated in a nitric oxide-independent manner. Further investigation is required to better understand this effect. Additionally, we observed that BTCI and PepChy promoted a negative inotropic effect in isolated hearts that was inhibited by the non-selective NOS inhibitor, L-NAME. The pre-treatment with L-NAME was not able to blunt the effects of PepTry on cardiac contractility, suggesting again, that the nitric oxide is not involved in the effects evoked by this peptide.

In conclusion, the presented results showed that BTCI, PepChy and PepTry promoted reduction in blood pressure and coronary vasodilation. These data indicate great therapeutic potential in the peptides, forming the basis for further studies into the development of new drugs for treatment of cardiovascular diseases.

## Material and methods

### Purification of BTCI and its derived peptides, PepTry and PepChy

BTCI was purified from *Vigna unguiculata* seeds as previously described by Ventura *et al*.^13,65^. In brief, crude extracts (CE) were obtained through incubation of homogenized seeds in distilled water in the presence of 10.0 μM phenylmethanesulfonyl fluoride (PMSF), a synthetic protease inhibitor, at 4 °C for 12 hours, followed by precipitation with 2.5% (v/v) TCA (trichloroacetic acid PA-C_2_HCl_3_O_2_) (Sigma Aldrich, USA) and 50% ammonium sulfate ((NH_4_)_2_ SO_4_) (m/v). The suspension was centrifuged at 8.000 g for 40 minutes at 4 °C and the precipitate (CE) collected, dialyzed and stored at 4 °C. Purification of BTCI was achieved with one step of ion exchange chromatography using previously activated DEAE-cellulose resin (Sigma Aldrich). The proteins were eluted using 10 mM phosphate buffer, pH 7.3 and a linear gradient of 0–800 mM NaCl. The fractions corresponding to BTCI were dialyzed and lyophilized.

PepTry (CTKSIPPQC-OH; S-S on Cys 1–9) and PepChy (CTFSIPAQC-OH; S-S on Cys 1–9) were purchased from AminoTech Research and Development (São Paulo, SP, Brazil), synthesized by a standard solid-phase method using the FMOC strategy^66,67^. FMOC-amino acids and reagents were purchased from NovaSyn TGA (Novabiochem, San Diego, CA, USA). Peptides were synthesized through steps of deprotection of the FMOC group using 20% (v/v) 4-methylpiperidine in dimethylformamide (DMF). The complete peptide linked to the resin was obtained after successive cycles of peptide bond formation using 2-(1H-Benzotriazole-1-yl)-1,1,3,3-tetramethylaminium tetrafluoroborate (TBTU) in DMF at room temperature and followed by removal of FMOC groups and coupling with the subsequent amino acids^68^. The peptide was cleaved from the resin with 92.5% (v/v) trifluoroacetic acid (TFA), 2.5% (v/v) thioanisole, 2.5% (v/v) 1,2-ethanedithiol, and precipitated using diisopropyl ether (−20 °C). The precipitate was solubilized using 50% (v/v) acetonitrile (ACN) and then lyophilized. The formation of disulfide bonds was achieved in diluted solution buffered adjusted with NH_4_OH at pH 8.

Peptides were purified by reverse-phase high performance liquid chromatography, RP-HPLC (Class LC-20A from Shimadzu Corp., Kyoto, Japan), using a semi-preparative C18 Shim-pak VP-ODS column (5 µm, 4.6 × 250 mm) (Shimadzu Corp., Kyoto, Japan) with a linear gradient (5–45%) of ACN. The column was washed with 95% ACN and 0.1% TFA and equilibrated with 5% ACN and 0.1% TFA. Crude peptide extract was applied to the column and subjected to a linear gradient (5 to 95%) of ACN for 90 minutes at a flow rate of 1.0 mL/min. The chromatographic profile was followed by reading the absorbance at 220 nm.

Purity and molecular mass of BTCI and peptides were estimated by electrospray ionization mass spectrometry (ESI-MS) on a LCMS-2020 system (Shimadzu Corp., Kyoto, Japan). The positive reflector with external calibration, as well as molecular mass range of calibrants (900–9100 Da), were considered. Concentration of the peptides was estimated using absorbance at 215, 225 and 205 nm, as follows^69^:

1 $$C\,(\mu g/mL)=\frac{[({A}^{215}-{A}^{225})x\,144]+({A}^{205}\,x\,31)}{2}x\,dilution\,factor$$

### Inhibition assay of BTCI, PepTry and PepChy against trypsin and chymotrypsin

The inhibitory activities of BTCI, PepTry and PepChy against trypsin and chymotrypsin activities were assayed using the substrates Na-benzoyl-DL-arginine-p-nitroanilide (BAPNA) and n-glutaryl-L-phenylalanine-p-nitroanilide (GPNA), respectively (Sigma)^19,39^. Enzymatic assays were performed in 50 mM Tris-HCl, CaCl_2_ 20 mM pH 7.6 for chymotrypsin or pH 8.2 for trypsin. Forty μL of BTCI and PepTry (0 to 20 μM) and BTCI and PepChy (0 to 50 μM) were incubated with 40 μL of trypsin (2.57 µM) or chymotrypsin (28.60 µM), respectively, in a 96-well plate at room temperature for 15 minutes. Then, 200 μL BAPNA (0.064 mg/mL) or GPNA (0.8 mg/mL) were added. The reaction was stopped by addition of 30 μL 30% acetic acid (v/v). Enzymatic hydrolysis of the substrate was evaluated by recording the absorbance at 410 nm. The residual activities of the enzymes, in the presence of inhibitors, were estimated considering the free enzyme activity to be 100%. Inhibition constants of the enzyme-inhibitor complexes, Ki, were calculated from fitted inhibition curves^70^ using the GRAFIT program version 3 (Erithacus Software, Horley, Surrey, United Kingdom).

### ACE inhibition assay

Inhibitory activities of peptides PepTry and PepChy and BTCI against ACE were performed according to Hayakari, *et al*. (1978) using the synthetic substrate hippuryl-L-histidyl-L-leucine (HHL)^71^. Briefly, ACE stock solution was obtained from rabbit lung in 10 mM sodium phosphate buffer, pH 8.3, and BTCI and peptides stock solutions were prepared in MilliQ water (m/v). Concentration of BTCI was estimated from spectrophotometric absorbance at 280 nm based ${A}_{280}^{1 \% }$ = 8.23. Concentration of PepTry and PepChy were estimated according to Eq. 1. BTCI and peptides were diluted in 10 mM sodium phosphate buffer pH 8.3 to obtain concentrations ranging from 0 to 600 µM. Ten µL peptide solutions were pre-incubated with 10 µL of ACE containing 0.5 M NaCl at 37 °C for 10 min. The mixture was then incubated with 10 µL substrate containing 12.5 mM HHL in 10 mM sodium phosphate buffer, pH 8.3 and 300 mM NaCl at 37 °C for 45 min. The enzymatic reaction was terminated by adding a mixture containing 550 µL of 100 mM sodium phosphate pH 8.3 and 332.5 µL of 3% (m/v) 2,4,6-trichloro-s-triazine (TT) (in dioxane). The mixture was incubated for 5 min. at room temperature and then centrifuged at 1000 *g* for 10 min at 4 °C. The absorbance values of supernatants containing hippuric acid liberated from HHL were recorded at 382 nm using the Jasco Spectrophotometer V-530 (Jasco Analytical Instruments, Tokyo, Japan). IC_50_ values for peptides and BTCI were calculated from nonlinear regression curve of ACE residual activity (%) *versus* inhibitor concentration (µM) (BTCI, PepTry and PepChy) using Origin software (OriginLab Corporation, USA). The control was prepared containing only the substrate without enzyme solution. Assays were performed in triplicate.

### Interaction of Peptides with Angiotensin converting enzyme (ACE) by docking procedure

The binding of peptides into the ACE active site was performed using the Evolutionary Algorithm for Docking (EADock)^72^, available at http://www.swissdock.ch/docking#. This algorithm is based on two adjust functions of molecular docking, interfaced with the CHARMM package for binding energy calculations and outputs the atomic coordinates of the protein-ligand complex. The tridimensional coordinates of target protein (ACE, PDB code 1O8A) and ligands, PepTry and PepChy, derived from BTCI structure (PDB code 3RU4), were considered for the docking procedure. The tridimensional coordinates of ligands were prepared using the Pymol program (DeLano Scientific LLC) and converted into a MOL2 file format with all hydrogen using the Open Babel GUI programa^73^ version 2.4.1. The best model was ranked according to the position of ligands closest to the ACE active site, as well as the docking scores (Full Fitness) and the most favorable estimated Gibbs free energy values for the ACE-peptide binding process.

### *In vivo* assays: hemodynamic and cardiovascular effects of BTCI, PepTry and PepChy

#### Animals

Adult male Wistar (WR) and spontaneously hypertensive (SHR) rats weighing 250 to 300 g from the Department of Physiological Sciences of the Federal University of Goiás, Brazil, were employed in all *in vivo* assays. The animals were housed in a special room under controlled conditions of a 12-h light–dark cycle and at 23 ± 1 °C, with free access to water and food. The use of animals and the protocols for *in vivo* and *ex vivo* assays were approved by the Ethics Committee of Animal Use and Care at the Federal University of Goiás, Brazil (protocol numbers 13/2016 and 039/2017).

#### Surgical procedures

Surgical procedures were performed as previous described^19^. Rats were anesthetized with isoflurane (2% in O_2_) and arterial blood flow (ABF) measurement was carried out from the right femoral or brachial artery via catheters. Administration of drugs was made through a catheter inserted into femoral veins. Anesthesia was maintained with urethane (1.2 mg.kg^−1^, iv.). Tracheotomy was performed in order to reduce respiratory effort and airway resistance. The electrocardiogram (ECG) was monitored from needle electrodes on the forelimbs. For recording hemodynamic parameters rats were placed in a stereotaxic apparatus (Insight Ltda., Ribeirão Preto, SP, Brazil) in the ventral decubitus position. Body temperature was maintained at 37 ± 1.0 °C with a heated water-circulating pad.

#### Hemodynamic and cardiovascular parameters recording

After stabilization of the cardiovascular parameters, 0.9% NaCl (vehicle - V) = or BTCI (30.0 mg/kg) and its derived peptides, PepTry and PepChy (3.3 mg/kg) were administered via gavage. The hemodynamic and cardiovascular parameters, systolic blood pressure (SBP), diastolic blood pressure (DBP), renal vascular conductance (RVC) and aortic vascular conductance (AVC) were recorded immediately after gavage and were monitored during 6 hours.

Pulsatile arterial pressure (PAP) measurements were performed through an arterial cannula connected to a pressure transducer linked to a specific amplifier (MLT0380 and Bridge Amp, ML221, AD Instruments, Bella Vista, Australia). Hemodynamic parameters were recorded (1000 samples/s), using a digital analog converter and LabChart 7 software (PowerLab 4/25, ML845, AD Instruments, Bella Vista, Australia). ECG signals were recorded (2.000 samples/s) using a digital analog converter, and were considered to calculate HR. The RBF and ABF were recorded in absolute values (mL/min) through a miniature probe on the left renal artery and abdominal aorta connected to a T206 flowmeter (Transonic Systems, Inc., Ithaca, NY, USA). The recorded signals (1000 samples/s) were analyzed using the PowerLab 4/25, ML845, AD Instruments, Bella Vista, Australia). The RVC and AVC were estimated by the ratio of RBF/mean arterial pressure (MAP) and ABF/MAP, respectively. The ΔRVC and ΔAVC were expressed as percentage change.

### *Ex vivo* assays: Effects of BTCI, PepChy and PepTry on left ventricular contractility and coronary vasodilation

Male WR and SHR weighing 250 to 300 g were employed as described above. To evaluate the cardiac effect of BTCI, PepChy and PepTry, isolated hearts were perfused according to the Langendorff technique^74^. Heparin (200 I.U) was injected intra-peritoneally and after 10–15 min animals were decapitated. The heart was carefully dissected and connected in the Langendorff system through the aortic stump and perfused with Krebs-Ringer solution containing 118.4 mM NaCl, 4.7 mM KCl, 1.2 mM KH_2_PO_4_, 1.2 mM MgSO_4_7H_2_O, 1.25 mM CaCl_2_2H_2_O, 11.7 mM glucose, and 26.5 mM NaHCO_3_. In addition, albumin (0.2 mg/mL) was added to Krebs Ringer’s solution to prevent aggregation of BTCI and peptides in the glassware of the system. The system was maintained with constant perfusion flow of 10.0 ± 2 mL/min, at 37 °C and constant oxygenation (5% CO_2_ and 95% O_2_). Isovolumetric left ventricular systolic and diastolic pressures were measured after insertion of a balloon into the cavity of the left ventricle through a left atrium incision. Diastolic intraventricular pressure was measured during coronary perfusion via a pressure transducer connected to the aortic cannula and was adjusted to 10 ± 2 mmHg. After 30 to 40 min, the hearts were perfused for 10 minutes with KRS containing (1) BTCI (1.0 nM), (2) PepChy (1.0 nM), or (3) PepTry (1.0 nM). To evaluate the possible effects of these peptides on the eNOS/NO pathway, hearts were perfused with KRS containing nitric oxide synthase inhibitor NG-nitro-L-arginine methyl ester (L-NAME; 1 µM) for 15 min before the perfusion of BTCI, PepChy or PepTr^61^y. The coronary vasodilatations induced by peptides were recorded and analyzed using a data acquisition system (DATAQ Instruments, Akron, OH, USA).

### Statistical analysis

The GraphPad Prism software (v 6.03) was employed to calculate cardiovascular parameters as mean ± E.P.M (Mean Standard Error). Variance (two-way ANOVA) and a subsequent Tukey’s post-test were used to analyze the SBP, DBP, RVC and AVC variations. The maximum response after administration of BTCI, PepChy and PepTry were analyzed using the Student’s t-Test. The results obtained for left ventricular pressure from isolated hearts were compared using two-way ANOVA and Sidak’s post-test. A value of *p* < 0.05 was considered to denote a significant difference.

### Ethical approval and informed consent

All procedures were performed in accordance with institutional guidelines for the use of laboratory animals of the institution and the protocols were approved by the Ethics Committee of Animal Use of the Federal University of Goiás (protocol numbers 13/2016 and 039/2017).

## Data Availability

All data produced during this work are available from corresponding authors on reasonable request.
